# VHL-dependence of EHHADH Expression in a Human Renal Cell Carcinoma Cell Line

**DOI:** 10.15586/jkcvhl.v11i1.322

**Published:** 2024-01-29

**Authors:** Julia Felicitas Pilz, Marinella Klein, Elke Neumann-Haefelin, Athina Ganner

**Affiliations:** Renal Division, Department of Medicine, Medical Center, University of Freiburg, Faculty of Medicine, University of Freiburg, Freiburg, Germany

**Keywords:** ccRCC, EHHADH, fatty acid metabolism, RCC4, VHL

## Abstract

The von Hippel-Lindau tumor suppressor gene (*VHL*) is mutated in up to 90% of clear cell renal cell carcinoma (ccRCC) cases, thus playing a key role in ccRCC pathogenesis. ccRCC can be classified as a metabolic disease in which alterations in fatty acid metabolism facilitate cancer cell proliferation. Enoyl-CoA hydratase and 3-hydroxyacyl CoA dehydrogenase (EHHADH) is an enzyme involved in peroxisomal fatty acid degradation. It is primarily expressed in renal proximal tubule cells, presumably the origin of ccRCC. Although *EHHADH* is still a relatively unexplored gene, it is known to be differentially expressed in several tumors. In this study, analysis of several databases revealed that EHHADH expression is downregulated in ccRCC samples compared to healthy kidney samples. Moreover, cell culture experiments were performed to investigate the relationship between EHHADH and VHL at the gene and protein level. qPCR and Western blot analyses using the human ccRCC cell line RCC4 revealed that EHHADH is expressed in a *VHL-*dependent manner. RCC4 cells reconstituted with *VHL* show significantly higher EHHADH mRNA and protein levels than *VHL*-deficient RCC4 control cells. These results indicate that the downregulation of EHHADH in ccRCC reported may be due to the loss of *VHL* function. This study is the first to molecularly characterize EHHADH, a key enzyme in peroxisomal ß-oxidation, in relation to *VHL*, suggesting a potential pathogenic interaction that is worthy of further investigation.

## Introduction

Estimating the global incidence of 36 cancers in 185 countries, kidney cancer ranks 16th, accounting for 2.2% of all new cancer cases (1). The majority of kidney cancers are renal cell carcinomas (RCC), with clear cell RCC (ccRCC) being the most common histological subtype (2). In up to 90% of sporadic ccRCC cases, the von Hippel-Lindau (*VHL*) tumor suppressor gene is biallelically inactivated (3).The *VHL* gene, therefore, plays a key role in the pathogenesis of ccRCC (4).

A hallmark of malignant cells is metabolic reprogramming, which involves changes in fatty acid metabolism (5, 6). Fatty acids are essential building blocks of various lipids and, as a source of energy, enable tumor cells to survive in nutrient-poor conditions (7). In addition, they are involved in cell signaling and, as a component of membrane lipids, are essential for rapid tumor proliferation (5, 6).

Since ccRCCs show genetic alterations in several metabolic pathways, this tumor can be regarded as a metabolic disease (8). Dysregulation of lipid metabolism in ccRCC was described as early as 1987 (9). Furthermore, there is good evidence that increased fatty acid synthesis in ccRCC correlates with higher tumor aggressiveness and poor prognosis (10, 11).

Enoyl-CoA hydratase and 3-hydroxyacyl CoA dehydrogenase (EHHADH) is a bifunctional enzyme that catalyzes two of the four reactions of the classical peroxisomal β-oxidation pathway (12). During peroxisomal β-oxidation, very long-chain fatty acids (VLCFs) are broken down into shorter ones (12). Besides the liver, *EHHADH* is primarily expressed in the kidney, more specifically in the cells of the proximal tubule (13, 14). These cells are thought to be the origin of ccRCC (15). Within the kidney’s tubular system, the proximal tubule is the portion with the greatest reabsorption capacity and, therefore, the highest energy demand (16).

*EHHADH* has not been extensively studied, but it shows differential expression in tumors compared to normal tissues, with increased or decreased levels depending on the type of cancer (17–20). For instance, it is downregulated in hepatocellular carcinoma but upregulated in osteosarcoma (17, 21–23). Beyond its differential expression, EHHADH is increasingly recognized for its pathogenetic and/or prognostic significance in cancers such as hepatocellular carcinoma, osteosarcoma, colorectal carcinoma, and pituitary adenoma (17–20, 24). In particular, in hepatocellular carcinoma, higher EHHADH expression is associated with longer survival, fewer recurrences, and lower pathological stage (18, 25).

Analyzing data from The Cancer Genome Atlas (TCGA), Clinical Proteomic Tumor Analysis Consortium (CPTAC), and The Human Protein Atlas (26–28), this study shows that EHHADH expression is reduced in ccRCC and correlates with patient survival. Furthermore, this study is the first to demonstrate, using cell culture experiments, that *EHHADH* expression depends on *VHL* in a human ccRCC cell line.

## Materials and Methods

### Antibodies

Primary antibodies used in this study include antibodies to EHHADH (sc-393123, Santa Cruz Biotechnology, 1:500 dilution), VHL (68547, Cell Signaling Technology, 1:1000 dilution), and γ-tubulin (T6557, Sigma-Aldrich, 1:4000 dilution). Secondary antibodies used include HRP-conjugated anti-rabbit (7074, Cell Signaling Technology, 1:5000 dilution) and HRP-conjugated anti-mouse antibody (P0447, Dako, Agilent Technologies, 1:10000 dilution).

### Cell culture

The cells used included RCC4 cells and RCC4+VHL cells. Both were provided by I. Frew and have been described previously (29). While RCC4 cells are *VHL*-mutated, RCC4+VHL cells re-express *VHL* (reconstitution of *VHL* by retroviral transduction). RCC4 and RCC4+VHL cells were cultured in Dulbecco’s Modified Eagle’s Medium (DMEM) supplemented with fetal bovine serum (FBS) to a final concentration of 10% and Geneticin 0.5 mg/mL.

### Quantitative western blot analysis

To quantify the levels of endogenous proteins of interest, RCC4 and RCC4+VHL cells were split in parallel and lysed in a lysis buffer containing Triton X-100 buffer [1% Triton X-100, 20 mM Tris (pH 7.5), 50 mM NaCl, 50 mM NaF, 15 mM Na_4_P_2_O_7_, 0.1 mM EDTA (pH 8.0)] supplemented with 0.25 mM PMSF, 2 mM Na_3_VO_4_ and cOmplete protease inhibitor cocktail tablet (Roche). Lysates were centrifuged (14,000 rpm, 15 min, 4°C) and total protein content was determined by the Bradford method. Equal amounts of proteins were fractionated by sodium dodecyl sulfate-polyacrylamide gel electrophoresis (SDS-PAGE), transferred, incubated with primary (anti-EHHADH, anti-γ-tubulin, and anti-VHL) and secondary antibodies, and visualized according to standard protocols. Films were scanned and protein bands were quantified using LabImage 1D L340 software. Endogenous EHHADH band densities were normalized to γ-tubulin.

### Quantitative real-time PCR

Total RNA was extracted from RCC4 and RCC4+VHL cells using the RNeasy Mini Kit (Qiagen). The SuperScript Kit (Invitrogen) was used for conversion to complementary DNA. Quantitative real-time polymerase chain reaction (qPCR) was performed using the Takyon No ROX SYBR 2X MasterMix dTTP blue Kit (Invitrogen). Primers were used at a concentration of 100 nM, and each reaction was performed in a final volume of 10 μL. qPCR was run on a LightCycler 480 instrument (Roche). Three technical replicates were performed for each biological sample. The corresponding cycle threshold (ct) values were averaged. For analysis of relative changes in *EHHADH* expression between RCC4+VHL cells and RCC4 cells (controls), the 2^-ΔΔ^^*Ct*^ method was used. Glyceraldehyde 3-phosphate dehydrogenase (*GAPDH*) served as a normalization control.

The following primers were used for the qPCR (all sequences are listed 5’ to 3’):

EHHADH primer_1: TCACAAACCTGATCCCTGGC and AGCAGCTATCCCTTCTCCCA, EHHADH primer_2: TTGGTGTTGTTGGCTTGG and TCTACAGCAATCACAGGAATC, GAPDH: CATTTCCTGGTATGACAA and GTCTCTCTCTTCCTCTTG, VHL: CACAGCTACCGAGGTCAC and TGAGAGATGGCACAA ATAATTCAG.

### Databases and analysis

Analysis of TGCA and CPTAC data was performed using the University of Alabama at Birmingham Cancer (UALCAN) data analysis portal (26, 30). Survival analysis was based on TCGA RNA sequencing (RNA-Seq) expression data and corresponding TCGA patient survival data. The latter included the number of days until the last follow-up for living patients and the number of days until death for deceased patients (30). Immunohistochemical imaging data for EHHADH expression was obtained from The Human Protein Atlas website [antibody HPA03640, kidney tissue sample: patient id1943 (27), renal cancer sample: patient id 2176 (28). Microsoft Excel, GraphPad Prism, and Adobe Illustrator were used for statistical analysis and graph preparation.

## Results

### EHHADH expression is downregulated in ccRCC and correlates with poor survival

**Figure 1:**
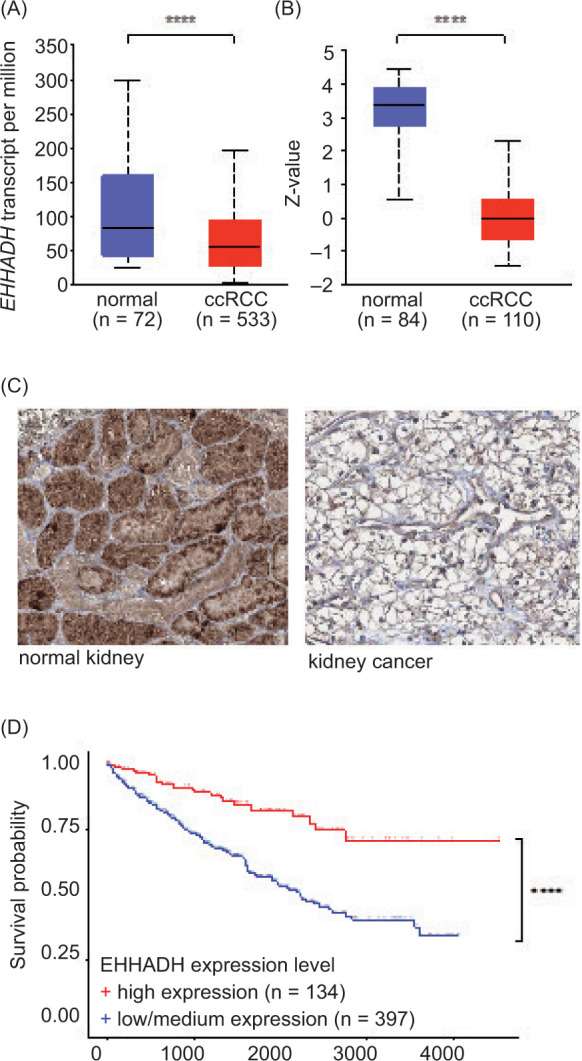
EHHADH expression is downregulated in renal cancer, and low *EHHADH* expression correlates with reduced survival. (A) *EHHADH* mRNA expression of primary ccRCC tumor samples compared to healthy renal control samples based on TCGA data. (B) *EHHADH* protein expression in primary ccRCC tumor samples versus healthy renal control samples based on CPTAC proteomics data. (C) Representative immunohistochemical staining with an *EHHADH* antibody from a kidney cancer tissue compared to a normal kidney tissue. (D) Correlation of *EHHADH* expression and patient survival probability based on TCGA primary tumor RNA-seq data and TCGA patient survival data. Primary tumor samples with high *EHHADH* expression were samples with transcripts per million (TPM) values equal to or above the third quartile, while samples with TPM values below the third quartile were described as low/medium expression. (A, B, D) Analysis was performed via the UALCAN website (26, 30). Data were taken from The Human Protein Atlas website (27, 28). (A, B) ****P < 0.0001 (t-test). (D) ****P < 0.0001 (log-rank test).

Analysis of TCGA data from the UALCAN website (26, 30) showed that *EHHADH* mRNA expression was significantly downregulated in primary ccRCC samples compared to healthy control kidney samples (Figure 1A). The CPTAC mass spectrometry-based tumor proteome dataset, also available on the UALCAN website (26), revealed that EHHADH protein levels were significantly lower in primary ccRCC tissue than in normal kidney tissue (Figure 1B). Representative immunohistochemistry data from The Human Protein Atlas (27, 28) consistently demonstrated that kidney tumor samples expressed lower levels of EHHADH compared to healthy kidney samples (Figure 1C). Survival analysis based on TCGA primary tumor RNA-sequencing (RNA-Seq) expression data and TCGA patient survival data (26, 30) showed that low/medium *EHHADH* expression correlated with poorer survival in ccRCC patients (Figure 1D).

In summary, analysis of several databases revealed that EHHADH mRNA and protein expression is lower in kidney tumor samples than in control samples, and that reduced *EHHADH* expression correlates with poorer survival.

### EHHADH mRNA and protein levels are upregulated in RCC4 cells expressing VHL

Having seen that EHHADH is differentially regulated in ccRCC, a tumor highly characterized by the loss of *VHL* function, the ccRCC cell line RCC4 (29) was used to investigate whether *EHHADH* expression depends on *VHL*. RCC4 cells are derived from human renal cell carcinoma and are *VHL*-mutated (29) qPCR and Western blot analysis were performed to compare EHHADH mRNA and protein expression between *VHL*-mutated RCC4 cells (controls) and an RCC4 subline reconstituted with *VHL*, here referred to as RCC4+VHL cells.

**Figure 2:**
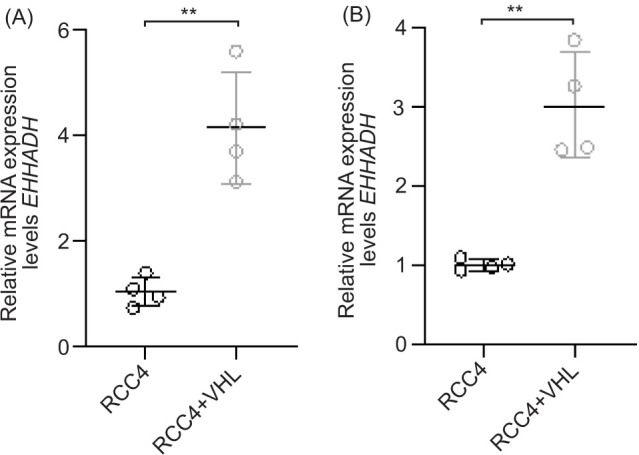
*EHHADH* mRNA levels are upregulated in the presence of VHL. qPCR analysis of relative *EHHADH* mRNA expression levels in RCC4 cells reconstituted with VHL (RCC4+VHL) compared to VHL-deficient RCC4 control cells (RCC4). Two different *EHHADH* primer sets, (A) primer_1 and (B) primer_2, were used. *EHHADH* expression levels were normalized to GAPDH. The four biological replicates of each experiment are shown as single values and mean +/- standard errors of the mean (SEM). **P < 0.01 (unpaired two-sample t-test).

qPCR analysis with two different *EHHADH* primer sets showed that RCC4+VHL cells had, on average, 4.2-fold and 3.0-fold higher relative *EHHADH* mRNA expression levels than RCC4 cells, respectively (Figure 2). The *VHL*-dependent difference in relative *EHHADH* mRNA expression levels was statistically significant.

**Figure 3:**
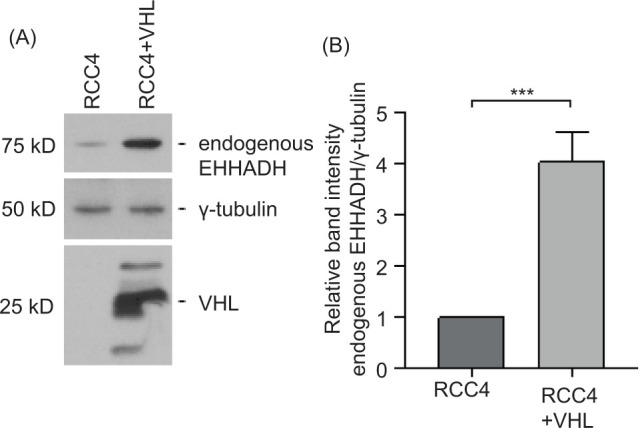
*EHHADH* protein levels are upregulated in the presence of VHL. (A) Cell lysates from RCC4 and RCC4 + VHL cells containing equal concentrations of total protein were analyzed by Western blot using anti-EHHADH antibody (upper panel), anti-γ-tubulin antibody (middle panel), and anti-VHL antibody (lower panel). γ-tubulin was used as a loading control. A representative Western blot of four biological replicates is shown. (B) Quantification of relative EHHADH levels normalized to γ-tubulin from the four independent experiments is shown. ***P < 0.001 (unpaired two-sample t-test). kD: kilodalton.

Moreover, cell lysates from RCC4 and RCC4+VHL cells were analyzed by Western blot using an anti-EHHADH antibody. RCC4+VHL cells showed a 4.0-fold higher relative band intensity of endogenous EHHADH than *VHL*-deficient RCC4 cells, indicating a significant difference in relative EHHADH expression between these two groups (Figure 3).

In conclusion, reconstitution of the *VHL* gene in RCC4 cells results in a significant upregulation of EHHADH mRNA and protein levels.

## Discussion

Database analysis revealed that EHHADH expression is reduced in ccRCC samples compared to normal kidney samples. Furthermore, low *EHHADH* expression is correlated with poor survival. This is consistent with previous reports, showing that enzymes involved in beta-oxidation, including EHHADH, are downregulated in ccRCC (31). However, to date, only one study by Xiao et al. has addressed the role of EHHADH in ccRCC in more detail (32). Using information from genetic databases and tumor tissue samples from 15 patients, Xiao et al. showed that EHHADH mRNA and protein expression is decreased in ccRCC compared to healthy kidney tissue (32). In addition, the EHHADH promoter is hypermethylated in ccRCC, indicating transcriptional silencing (32). Higher expression and lower methylation of EHHADH were associated with longer survival of ccRCC patients and lower pathological tumor stage (32).

The cell culture experiments described in this study showed that reconstitution of the *VHL* gene in the human ccRCC cell line RCC4 results in significantly higher mRNA and protein expression of EHHADH, indicating that EHHADH levels are regulated by *VHL*. EHHADH plays an important role in the β-oxidation of very long-chain fatty acids (VLCFs) in peroxisomes and is predominantly expressed in hepatocytes and proximal tubule cells of the kidney, the cells of origin of ccRCC (12–14). Although several reports have shown that cancer cells require β-oxidation, especially under stress conditions (6), for ccRCC a grade-dependent inhibition of β-oxidation has been described (31).

For hepatocellular carcinoma, it has been shown that a reduced number of peroxisomes and decreased peroxisomal fatty acid oxidation correlate with dedifferentiation of liver cells (33, 34). Moreover, in clear cell hepatic carcinomas, peroxisomes were found at the cell periphery (33). In the kidney, peroxisomes are usually highly abundant in renal proximal tubule cells and peroxisome abundance is reduced in a HIF-2α dependent manner, especially in well-differentiated grade 1 ccRCC and to a lesser extent in grade 2 and 3 tumors (35). This observation suggests that in addition to the HIF-2α-driven regulation of peroxisome homeostasis, other mechanisms, such as *VHL*-dependent EHHADH regulation, may also contribute to impaired peroxisomal β-oxidation in highly dedifferentiated ccRCC cells. Despite their huge significance for cell metabolism, peroxisomes’ functional effects in cancer are less understood than those of other metabolic organelles (36). This highlights the need to further analyze the impact of *VHL*-dependent EHHADH regulation on peroxisomal function and β-oxidation in the context of ccRCC dedifferentiation in more detail.

It has also been shown that the potent tumor suppressor p53 promotes the expression of enzyme genes involved in peroxisomal fatty acid β-oxidation, including EHHADH, thereby repressing purine biosynthesis and mediating tumor suppression in colorectal cancer (37). This occurs via the acetylation and subsequent inhibition of 5-aminoimidazole-4-carboxamide ribonucleotide formyltransferase/IMP cyclohydrolase (ATIC), a bifunctional enzyme that catalyzes the last two steps of de novo purine biosynthesis (37). Whether a similar mechanism could also apply to *VHL*-dependent EHHADH regulation and ccRCC remains to be clarified. Overall, the role of EHHADH and peroxisomal fatty acid ß-oxidation in ccRCC is still relatively unexplored, underscoring the importance of further analysis.

A major limitation of this study is that only preliminary hypotheses are presented, highlighting the need for validation through larger-scale research. Examination of *VHL* dependence on EHHADH expression was confined to a single ccRCC cell line, suggesting the necessity to include additional cell lines for broader applicability. Moreover, the use of third-party databases and analytical tools implies a dependency on external data quality, which may affect the reliability of conclusions. Despite strict adherence to standardized protocols and transparent reporting, a degree of inherent investigator bias may inevitably persist.

This study is the very first to molecularly characterize how *EHHADH* and *VHL* are related in ccRCC. Rather than providing a complete picture of the role of *EHHADH* and *VHL* in ccRCC, the intention is to suggest a potential pathogenetically relevant relationship worthy of future investigation.

## Conclusion

In summary, the results obtained here show that EHHADH mRNA and protein expression are reduced in primary ccRCC samples compared to healthy kidney samples. Reconstituting *VHL* in a ccRCC cell line leads to increased mRNA and protein levels of EHHADH, implicating that downregulation of EHHADH in ccRCC is due to the loss of *VHL* function.
